# Intimacy in Phone Conversations: Anxiety Reduction for Danish Seniors with Hugvie

**DOI:** 10.3389/fpsyg.2016.00537

**Published:** 2016-04-19

**Authors:** Ryuji Yamazaki, Louise Christensen, Kate Skov, Chi-Chih Chang, Malene F. Damholdt, Hidenobu Sumioka, Shuichi Nishio, Hiroshi Ishiguro

**Affiliations:** ^1^Hiroshi Ishiguro Laboratories, Advanced Telecommunications Research Institute InternationalKyoto, Japan; ^2^Section for Aesthetics and Culture, Department of Aesthetics and Communication, Aarhus UniversityAarhus, Denmark; ^3^Section for Global Studies, Department of Culture and Society, Aarhus UniversityAarhus, Denmark; ^4^Interdisciplinary Nano Science Center, Aarhus UniversityAarhus, Denmark; ^5^Department of Philosophy and the History of Ideas, Institute for Culture and Society, Aarhus UniversityAarhus, Denmark; ^6^Unit for Psychooncology and Health Psychology, Department of Psychology, Aarhus UniversityAarhus, Denmark; ^7^Department of Systems Innovation, Graduate School of Engineering Science, Osaka UniversityToyonaka, Japan

**Keywords:** telecommunication, Hugvie, anxiety, stress, personality

## Abstract

There is a lack of physical contact in current telecommunications such as text messaging and Internet access. To challenge the limitation and re-embody telecommunication, researchers have attempted to introduce tactile stimulation to media and developed huggable devices. Previous experiments in Japan showed that a huggable communication technology, i.e., Hugvie decreased stress level of its female users. In the present experiment in Denmark, we aim to investigate (i) whether Hugvie can decrease stress cross-culturally, i.e., Japanese vs. Danish participants (ii), investigate whether gender plays a role in this psychological effect (stress reduction) and (iii) if there is a preference of this type of communication technology (Hugvie vs. a regular telephone). Twenty-nine healthy elderly participated (15 female and 14 male, *M* = 64.52 years, *SD* = 5.67) in Jutland, Denmark. The participants filled out questionnaires including State-Trait Anxiety Inventory, NEO Five Factor Inventory (NEO-FFI), and Becks Depression Inventory, had a 15 min conversation via phone or Hugvie and were interviewed afterward. They spoke with an unknown person of opposite gender during the conversation; the same two conversation partners were used during the experiment and the Phone and Hugvie groups were equally balanced. There was no baseline difference between the Hugvie and Phone groups on age or anxiety or depression scores. In the Hugvie group, there was a statistically significant reduction on state anxiety after meeting Hugvie (*p* = 0.013). The change in state anxiety for the Hugvie group was positively correlated with openness (*r* = 0.532, *p* = 0.041) as measured by the NEO-FFI. This indicates that openness to experiences may increase the chances of having an anxiety reduction from being with Hugvie. Based on the results, we see that personality may affect the participants’ engagement and benefits from Hugvie. We discuss the implications of the results and further elaborations.

## Introduction

How can interpersonal communication media shape our social connections? People are engaged in communication through computer screens, tablets, and cell phones to the extent that every day human contact is turning digital. People express concern that our communication has become increasingly shallow and we forget to spend time in the natural human way, together with other humans. Body-contact such as hugging and face-to-face interactions is outsourced to global networks like Skype, Facebook, and Twitter. For some, the digitalization of human contact can be a threat to personal relationships, because of the lacking face-to-face contact. Yet at the same time, such media offer the promise of more opportunity for connection with more people, and others find benefits with new connections and stronger bonds through new communication media (Baym, 2010). In both perspectives, communication media are regarded to change the nature of our interpersonal connections.

The development of telecommunication technologies has given us many beneficial opportunities to communicate with people worldwide. With the start of phones, we were able to talk with people who were not in our presence. To encounter the spatial and temporal flexibility, new technologies such as internet and smartphones have been developed and widely used as preferred communication tools, because it improved people’s ability to stay in touch at anywhere and anytime, but information we can send and receive is typically limited to text based messages and video.

This means that tactile stimulation is still absent, so recent researches are attempting to introduce intimacy in form of physical contact to remote communication media by introducing wearable devices like “HugMe” (Cha et al., 2009) and robots to assist people in everyday life to facilitate social interactions. The HugMe is an interpersonal haptic teleconferencing system. By using it, the passive users like children with the haptic jackets are to feel the “touch” from the active users like their parents in remote. An example of robotic communication medium is a human-like robot “Telenoid” for telecommunication (Ogawa et al., 2011). It has a huggable design and can be used as welfare technology to enhance the social interactions of seniors, especially those who are cognitively impaired in order to remotely communicate with the appeal of intimacy, i.e., close relationship in embodiment. For those who have been affected by the digital divide, embodied communication technology like the Telenoid robot can provide an easy and attractive way to remotely communicate with others, and promote social interactions in both verbal and non-verbal ways. Recent attempts to re-embody the internet, with help from robotics, has left a question of determining in what way aspects of physical contact could be optimal conditions for communication (Seibt and Nørskov, 2012). In the line of these attempts, we explore the efficacy of telecommunication media that provide physical contacts and that acceptance might differ in different environments and culture.

Touch is one of our first senses and is our most fundamental means of contact with the world (Barnett, 1972). Studies demonstrate that interpersonal touch plays a crucial role in the development and well-being of humans (Field, 2001). For example, the simple act of touching a patient by a nurse can result in a decrease in the patient’s level of stress (Whitcher and Fisher, 1979), and those infants whose mothers use more stimulating touch are reported to have better visual-motor skills (Weiss et al., 2004). Tactile sensations can have powerful effects on people’s behaviors and emotions, and facilitate bonding between pairs in a couple or groups in both animals and human (Boccia, 1986; Light et al., 2005). However, tactile aspects of communication are lacking in long-distance interactions as in phone conversation. Intimacy here can be defined as having a feeling of close relationship with others, for example, talking to your loved one through phone can be heartfelt and we ask how we can realize intimacy in phone conversation as or more than in face-to-face interactions. Due to the limitations of interpersonal touch in communication devices, new communication tools like wearable devices (e.g., google glass) have been designed to provide the opportunity to add physical contact to internet and phone users so that distance no longer would be a limitation (Bonanni et al., 2006). Also, researchers have been attempting to introduce assisting robots as communication tools to achieve psychological effects by physical contact (Kanda et al., 2002; DiSalvo et al., 2003; Stiehl and Breazeal, 2005).

Pet-like social robotic companions are introduced in elderly care, such as the small therapeutic seal-typed robot called “Paro.” The usage of Paro in nursing homes demonstrated a sense of companionship and decreased loneliness (Wada et al., 2005; Wada and Shibata, 2006). Media technologies have the potential for promoting communication and having positive psychological effects on elderly, even though many elderly are part of the group, who has trouble keeping up with new technologies. Paro promotes a feeling of comfort when touched, almost like a living animal, which are reported to have a therapeutic effect on stress both mentally and physically. In relation to this, studies have shown that touches, hugs and massages from people, even animal-assisted therapy, has a physiological effect on stress reduction (Beetz et al., 2012; Morhenn et al., 2012).

Many studies have reported endocrine responses to psychological stress and stressful tasks, such as public speaking and mental arithmetic, can increase cortisol levels (e.g., Kirschbaum et al., 1993). Cortisol, known as the stress hormone, is produced in the adrenal glands and regulates many processes that occur in the body in response to stress within an effort to maintain homeostasis. Reviews have highlighted the effects of psychological stressors on this physiological system are variable and inconsistent (e.g., Biondi and Picardi, 1999). However, assessment of cortisol in blood and saliva to see psychological stress levels is a widely accepted and commonly used method in psychoneuroendocrinology (Kirschbaum and Hellhammer, 1994; Hellhammer et al., 2009).

In a previous experiment in Japan, a significant reduction in cortisol levels was shown for those who had conversations through the huggable communication medium Hugvie (Sumioka et al., 2013). However, the study had several limitations. Gender, age, and cultural background could all influence the endocrine changes and modulate people’s interpretation of, and hence their response to, interpersonal touch, as reported (Gallace and Spence, 2010). The Japanese culture does not belong to the cultures in which people often touch each other, e.g., handshakes and hugs (Finnegan, 2005). The interpersonal touch is a cultural phenomenon, therefore in Japan the custom of bowing and limiting touches could have affected the result of the previous study, as the participants might, due to the lack of body contact, have reacted excessively resulting in overstimulation while hugging the communication media.

Culturally Europeans such as Danes have a longer history with body contact, hugs and handshakes, in everyday life. The societal openness to new technologies makes Denmark ideal for testing Hugvie on a cross-cultural and gender basis. In the experiment in Japan, all the participants were female, which therefore did not allow for exploring whether decrease in cortisol level was due to gender differences. Therefore, the results of the Japanese experiment needs to be replicated and tested in other social settings. In the Danish experiment, we aim to investigate if (1) the decrease of stress when using Hugvie is different or the same between Japanese and Danish participants as well as (2), investigate whether reduction of the cortisol level and preference for this type of communication technology is influenced by gender (by including males in the study).

Hence the purpose of this experiment is to investigate (i) whether Hugvie can decrease stress cross-culturally, i.e., Japanese vs. Danes, (ii) investigate whether gender plays a role in this psychological effect (stress reduction) and (iii) if there is a preference of this type of communication technology (Hugvie vs. a regular telephone).

## Materials and Methods

In this experiment, we replicate a previous experiment in Japan by using a similar method, so in this present study the human-shaped Hugvie pillow-phone was compared with a regular phone. We setup almost identical experimental conditions as described in the following article: “Huggable communication medium decreases cortisol levels” (Sumioka et al., 2013). The study by Sumioka et al. (2013) found strong correlations between saliva and blood cortisol levels. Therefore, in our experiment, we only took saliva samples, as blood samples would be redundant.

Both male and female participants were equally and randomly divided into two groups who all had to talk with the same stranger of opposite gender while either hugging Hugvie (Hug Group) or talking in a regular mobile phone on speaker (Phone Group). The latter was the control group. To evaluate participants’ psychological and physiological responses to the social interaction through the communication medium, we measured cortisol levels through saliva samples and had the participants answer questionnaires at baseline and after the conversation session. The participants were video-recorded during the session in order to follow and evaluate their reactions and behavior in comparison with cortisol level. In addition, we decided to interview the participants after the sessions to see how they perceived the communication media.

As the results were positive for the Japanese women in the previous study, we predicted that we would come to a similar result, but with gender differences. For the purpose of the psychological effect, we evaluated the questionnaires outcome and expected to find changes in answers after the sessions in the Hugvie group. We assumed personality traits, as well as cultural background, might be related to the result.

An ethical committee in Jutland, De Videnskabsetiske Komiteer, For Region Midtjylland decided that an approval was not needed for this experiment. It was also checked and approved by the committee at Cognition and Behavior Lab, Aarhus University.

### Communication Device

**FIGURE 1:**
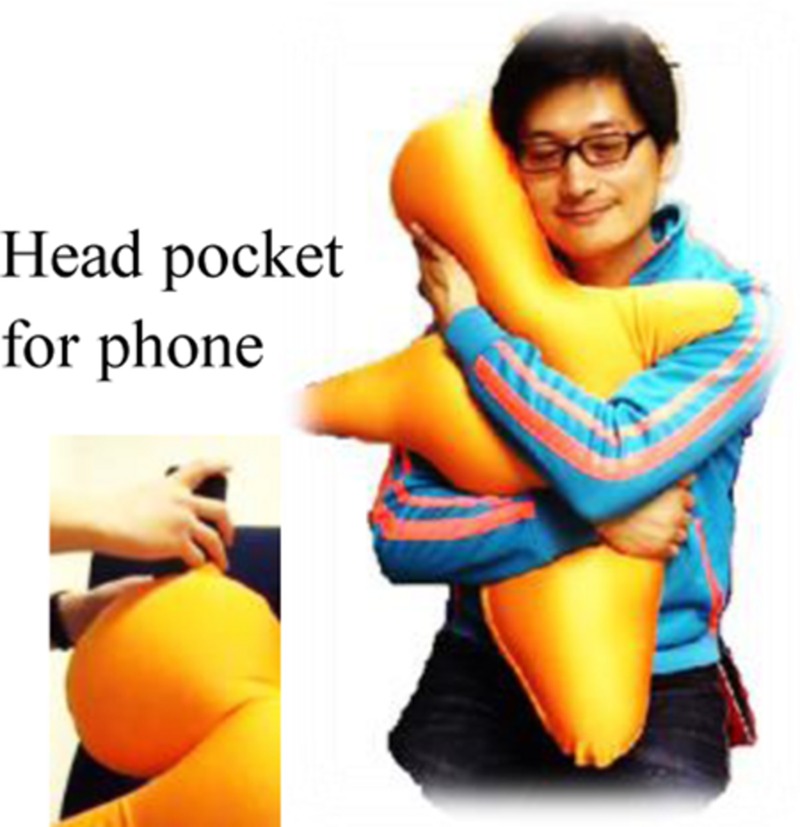
**Hugvie**.

The Hugvie^®^ (**Figure 1**) was developed by Osaka University and ATR Hiroshi Ishiguro Laboratory. It is a pillow-phone in a minimalistic human form for talking whilst hugging. Its height is 75 cm and its weight is 600 g. It is designed to enable users to feel the presence of any remote partners strongly while communicating with them. The human-like robot with minimalistic characteristical traits called the Telenoid (Ogawa et al., 2011), was the inspiration for creating the Hugvie pillow-phone.

In studies with the Telenoid it was reported that physical contact, e.g., hugging, has an impact on the psychological state of the user (Ogawa et al., 2011). In line with these findings, the Hugvie was designed with focus on the hugging experience. Its pillow like feeling stems from the spandex fiber cover with polystyrene microbead filling. Like the Telenoid, there are no actuators inside it, Hugvie appears like a person with open arms ready for a hug. With a pocket design, it is possible to place a mobile phone inside its head. This is intended to give the user a feeling of hugging their conversation partner while talking through the pillow. Because of its design, it is possible to investigate the effect of touches.

### Subjects

The experiment included totally 29 healthy participants (15 female and 14 male). They were elderly healthy subjects (fine elder citizens; *M* = 64.52 years, *SD* = 5.67), who were invited to evaluate our communication media. We used flyers and posters, which were distributed throughout the city in places like activity centers and libraries where elderly people gather. We also asked staff at elderly care centers and officials with broad networks within the senior community to help gather participants. We targeted elderly because they have a higher hormonal stability than younger people do, especially in the case of women. Exclusion of participants would happen in case of acute or chronic hormonal dysregulation or if they were on any kind of hormonal medication. The participants received oral and written information about the study and gave their written informed consent. Furthermore they were informed that the experiment included several prohibitions such as alcohol intake or smoking 1 day ahead of the study and to refrain from drinking, eating, or exercising 1 h before the session. The Hug and Phone groups were randomly selected, yet evenly spread in morning and afternoon sessions. They were not informed prior to the session of which group they were assigned to.

### Conversation Partner

We selected two capable conversation partners among students at Aarhus University. They proved to possess good conversational skills and could with ease fill out 15 min of conversation in the experiment. As in the previous experiment in Japan, the conversation partners were university students in their 20 s a, but not only a male (27 years old) but also female (28 years old). They received basic information about the experiment and gave informed consent in the same way as the participants.

### Experimental Environment

**FIGURE 2:**
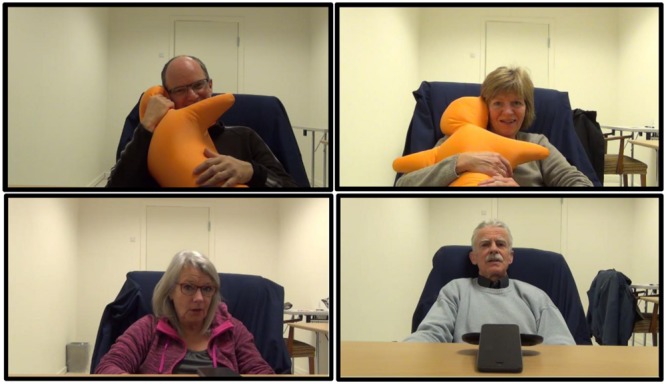
**Experimental settings**.

The experiment took place at COBE Lab at Aarhus University. The participants filled out the questionnaires and had the conversation in separate rooms. In the questionnaire room, we prepared and accepted the informed consent, the pre-conversation questionnaires and one of our staff assisted with the first saliva sample in this room. In the conversation room, we prepared a big cozy chair, a camera, post-conversation questionnaires and the second saliva sampling tube. We brought either the phone or Hugvie depending on the participant’s group right before the conversation session started (**Figure 2**). The participants never met their conversation partners, who made the phone calls from another room. During the conversations and the questionnaires, participants were left alone.

### Experimental Procedure

The experiments were conducted evenly in the morning and the afternoon (8:00–12:00 and 13:00–17:00) in according to the Japanese experiment. After filling out pre-conversation questionnaires about their feelings of anxiety, stress level, and personality by using questionnaires the State-Trait Anxiety Inventory (STAI), Becks depression inventory (BDI-II), Geriatric Depression Scale (GDS), Perceived Stress Scale (PSS), and NEO Five Factor Inventory (NEO-FFI), saliva samples were given by all participants.

After this, the participants came to the conversation room and could relax for about 5 min before they were given either the Hugvie or phone on speaker. All participants conducted a 15-min conversation with the conversation partner of opposite sex with the given communication media. The conversation partner has prior been informed to introduce himself/herself and ask for the participants’ name, where after they ask the participant about their best memories of the past year. It was allowed to have free conversation during the session with the exception of questions about their educational background, parents’ jobs, and political views for ethical reasons. After the conversation, the second saliva sample was retrieved and the participant was asked to fill out the post-conversation questionnaire about their feelings of anxiety throughout the conversation and finally we conducted a brief interview to hear the participants’ opinion about the conversation and media usage.

### Questionnaires

We asked the participants about their feelings in the pre- and post-conversation questionnaires. For the pre-conversation questionnaires, we used the STAI, the PSS, the BDI-II, the GDS, and the NEO-FFI. For the post-conversation questionnaires, we used the STAI and PSS. The STAI is a commonly used measure of trait and state anxiety (Spielberger, 1983). It consists of 40 questions and differentiates between the temporary condition of state anxiety and the more general and long-standing quality of trait anxiety. We used all the questions of the STAI before the conversation-session and repeated only the state part after the session. To obtain subjective stress measure, we also used the 10-item PSS that is a measure of the degree to which situations in one’s life are appraised as stressful (Cohen et al., 1983).

We used the GDS and BDI-II only at baseline to ensure clinical depression did not interfere with results. To assess the level of depression in elderly, we used the BDI-II composed of 21 items (Beck et al., 1996) and a short version of the GDS containing 15 questions with simple yes/no response set (Sheikh and Yesavage, 1986). To investigate the relations between the participant’s personality and the changes in stress level, we also used the 60-item NEO-FFI that provides a concise measure of the five basic personality factors (Costa and McCrae, 1989).

The NEO-FFI was used to assess five stable personality dimensions as derived from the five-factor model of personality (NEO-PI-R). The NEO-PI-R is validated cross-culturally (McCrae, 2002) and is available in a validated Danish version. The NEO-FFI items were administered verbally and the respondents rated them on a five point Likert scale from “strongly disagree” to “strongly agree.” The five personality dimensions are Openness (openness to internal and external stimuli), Conscientiousness (self-discipline and competency), Extraversion (tendency to be sociable and adventurous), Agreeableness (degree of trustfulness, modesty), and Neuroticism (tendency toward experiencing psychological distress or negative affect).

### Cortisol Collection and Analysis

The saliva samples were assayed for cortisol determination by a cortisol enzyme immunoassay (Cortisol EIA; Arbor Assays, USA) using a standard curve method with reported detection limits of 45.4 pg/ml. The assay was performed as instructed by the manufacturer. The cross-reactivity of the assay is 18.8% with dexamethasone, 1.2% with cortisone, 7.8% with prednisolone, 1.2% with corticosterone and <0.1% with progesterone. Saliva was obtained at least 2 h after eating. The participant’s mouth was rinsed prior to saliva collection to avoid food borne antigens or other materials that may affect cortisol analysis.

Once the saliva was collected, protease inhibitors (Complete protease inhibitor cocktail tablets, Roche) were added according to the manufacturer’s protocols to prevent protein degradation and it was stored at -80 until the assay. For the saliva assay, thawed samples were centrifuged at 2500 *g* for 20 min and the supernatant was collected for the assay. All samples from the participants were included in the same assay batch to eliminate within subject inter-assay variance. All the samples were assayed in duplicates and averaged. The effect of physical touch was measured as the decrease of cortisol that was calculated by subtracting the cortisol levels before the conversion from those after the conversion.

## Results

Data was analyzed using IBM SPSS Statistics for Macintosh, Version 21.0. (2012; Armonk, NY: IBM Corp). Paired and un-paired two-tailed *T*-tests were used for group comparisons on continuous variables as this statistical procedure have been found to be robust even in very small samples (de Winter, 2013). Bonferroni adjustments were not applied. The relationship between personality traits and changes in anxiety level was explored by Spearman correlations.

There was no significant age difference between the Hugvie (*M* = 64.9, *SD* = 6.4) and Phone groups (*M* = 64.1, *SD* = 5.2), *t*(27) = 0.396, *p* = 0.695. There was no significant baseline differences in reported depression as assessed by Becks Depression Inventory between the Hugvie (*M* = 4.2, *SD* = 4.69) and phone group (*M* = 2.42, *SD* = 2.56), *t*(27) = 0.223, *p* = 0.176.

Paired samples *t*-test showed no statistically significant differences between baseline (*M* = 8.50, *SD* = 4.23) and post-encounter scores (*M* = 9.21, *SD* = 4.37) on the Perceived Stress Scale for the Hugvie group, *t*(13) = -1.046, *p* = 0.315 or for the Phone group (Baseline: *M* = 9.21, *SD* = 3.47; Post-encounter: *M* = 9.14, *SD* = 3.99), *t*(13) = -0.126, *p* = 0.902.

Paired samples *t*-test showed a statistically significant difference between baseline (*M* = 32.55, *SD* = 6.4) and post-encounter scores (*M* = 27.86, *SD* = 4.53) on STAI for the Hugvie group, *t*(14) = -3.362, *p* = 0.005 but not for the Phone group (Baseline: *M* = 28.78, *SD* = 3.42; Post-encounter: *M* = 28.07, *SD* = 3.35), *t*(15) = -0.924, *p* = 0.372.

A STAI change score was calculated (Baseline STAI score minus post-encounter STAI scores) and correlated using a Spearman correlation to personality traits as assessed with the NEO-P-IR (for descriptives of personality traits for the Phone and Hugvie groups see **Table 1**). In the Hugvie group (*n* = 15) there was no significant correlation between STAI change score and extraversion (*r* = 0.151, *p* = 0.591), agreeableness (*r* = 0.213, *p* = 0.446) or neuroticism (*r* = 0.432, *p* = 0.108). However, there was a positive correlation with openness (*r* = 0.532, *p* = 0.041) and a near-significant correlation with conscientiousness (*r* = -0.509, *p* = 0.053). For the phone group (*n* = 14) there was no correlations between anxiety state changes and any of the personality traits: extraversion (*r* = 0.156, *p* = 0.595), conscientiousness (*r* = -0.050, *p* = 0.864), agreeableness (*r* = -0.064, *p* = 0.836) neuroticism (*r* = 0.98, *p* = 0.739) or openness (*r* = 0.257, *p* = 0.397).

**Table 1:** Description of personality traits for Phone and Hugvie groups.

	Phone group	Hugvie group
	(*M, SD*)	(*M, SD*)
Openness	31.54 (8.11)	32.20 (4.16)
Conscientiousness	36.57 (3.90)	31.93 (5.04)
Extraversion	32.50 (5.24)	31.73 (5.85)
Agreeableness	36.31 (4.05)	34.20 (4.60)
Neuroticism	13.50 (5.92)	15.73 (6.53)

A Chi-square test for independence with Yates Continuity Correction indicated that there was no significant association between whether the participant was assessed in the morning or afternoon and which group he/she was ascribed to (Hugvie or phone group), *X*^2^(1, *N* = 29) = 0.03, *p* = 0.87, phi = -0.100. There was no significant difference in cortisol level from baseline to post-encounter for the Hugvie (Baseline: *M* = 1.86 ng/ml., *SD* = 0.95; Post-encounter: *M* = 1.70 ng/ml., *SD* = 0.96) *t*(13) = -1.01, *p* = 0.330 or the phone group (Baseline: *M* = 1.79 ng/ml., *SD* = 1.18; Post-encounter: *M* = 1.63 ng/ml., *SD* = 0.89) *t*(12) = 1.96, *p* = 0.457 using paired samples *t*-test.

A cortisol change score was calculated (post-encounter cortisol level minus baseline cortisol level) and using a Spearman correlation it was correlated to the STAI change score. There was no significant correlation with STAI change score in the Hugvie group (*r* = 0.043, *p* = 0.884) or the phone group (*r* = -0.077, *p* = 0.803) nor was cortisol change correlated significantly to changes in perceived stress score for the Hugvie group (*r* = -0.436, *p* = 0.136) or phone group (*r* = 0.443, *p* = 0.130).

## Discussion

The purpose of this paper is to describe how the huggable communication medium Hugvie could be perceived and effective among Danish people. In the Hug group there was a significant difference between baseline and post-encounter scores and we found that Hugvie is effective in reducing anxiety for Danes as well cross-culturally. No significant differences were found in the phone group. Another finding was that the difference was related to personal traits, namely openness. There was also a near-significant correlation between STAI change score and conscientiousness. There was no gender difference to see in the questionnaires.

The cortisol level showed null-results which could be a lack of sensitivity of the cortisol measures or even reactivity in light of cultural differences. From a previous study, the cortisol level was increased with a lesser extent compared with that of amylase after participants were exposed to a stressful video (Takai et al., 2004). It took longer for the cortisol to show changes than amylase, so the sessions for our Hugvie experiment might not have been long enough to make use of cortisol and it might have been better to include changes in amylase levels too. Therefore, we will focus our discussion on the questionnaire results and interviews.

### Personality Matters

The relation between anxiety reduction and openness as seen in the questionnaire results could indicate that users with sensitivities, such as openness to new experiences, would be the main group among Danes to benefit by using the huggable medium. If the user, for example, would have an active imagination and would be sensitive to aesthetics, Hugvie could be helpful in reducing anxiety and hereby stress. The greater likelihood of experiencing an anxiety reduction when using Hugvie would therefore be, in this case, elderly who score higher on openness.

We did not see any positive outcome with other personalities in this experiment, but on the other side, there was also a near-significant negative correlation between anxiety state changes and conscientiousness in the Hug group. This indicates that people who are high on conscientiousness have a greater likelihood of becoming anxious with Hugvie. It has been found that conscientiousness is negatively related to creativity; whereas, openness to experience relates to it positively (George and Zhou, 2001). We see these personality traits affect on the effects of Hugvie in both ways, positively and negatively, thus, could be an important factor for media usage.

Openness to experience is a personality trait in the five factor model of personality and is fundamental for aesthetic appreciation and creativity. Openness consists of a set of specific tendencies that cluster together, involving six facets like imagination. A model of openness divides the trait into the two groups, i.e., openness and intellect (DeYoung et al., 2007). The openness aspect is about the heart that includes aesthetic sensitivity, creativity, and imaginativeness while the intellect aspect is a brain division that includes fluid intelligence, vocabulary knowledge, and an intellectual life approach.

Openness to experience has consistently predicted aesthetic appreciation and engagement in the arts where people immerse in aesthetic activities such as reading, painting, visiting art galleries, and valuing the arts (McManus and Furnham, 2006). People with high openness draw more enjoyment from, have more positive attitudes toward, and are gladly more exposed to the arts (Fayn and Silvia, 2015). Exposure to new media can for some people be joyful and beneficial whilst others cannot gain the same experience. This can be seen in parallel with aesthetics in artifacts as a singular experience meaning the experience that differs according to time, place, mood, and so on.

We consider the idea of aesthetic experience as a singular experience. Every meeting or experience with an artifact, for instance a piece of art, like a painting is singular, and it differs from individual to individual how the artifact is experienced; the individual’s state of mind on that specific day, as well as the setting. If you see a painting more than once you will never have the same experience thereby making it singular. Maybe you were in a different mood, maybe you were with different people, or you see the painting a different place than the first time.

This theory is used in situations when meeting aesthetic artifacts like artwork. Furthermore, there is the idea that you have to be open to the aesthetic potential or value of an artifact to experience it as an aesthetic artifact. If you are not open to the artifact’s aesthetic potential, you cannot have an aesthetic experience. Therefore, it can also be that having an open mind to Hugvie and the very experience is necessary. You have to be open to accept the way holding Hugvie makes you feel. For instance, one female participant did not like holding Hugvie because she felt she had outgrown soft toys, while a male participant started feeling it being natural to hold Hugvie quite fast. It was a positive experience for some participants, but also an utterly negative experience for a few, while some did not really think about and were indifferent to it. It could therefore mean that some of the participants were not even very open to the idea in the first place or it could also mean that they just had a negative experience though still understanding that Hugvie could be helpful for others.

There were instances where people were skeptical and did not like the idea of Hugvie prior to the session based on the information about Hugvie provided in the flyers and posters. After the session, there was a significant anxiety reduction as a result and some even changed their opinions. However, only short experiments were conducted, so there is a possibility that the novelty of the medium attracted participants, and if they use it in a longer term the effect of reducing anxiety might disappear. To reduce Hugvie’s novelty effects, a longitudinal study is necessary for establishing its efficacy thoroughly. It will also be required to test it in settings that are more naturalistic like home for people to act freely.

### Social Norms and Media Usage

If I limit to statistically significant results reported in this paper, I can find no evidence for cross-cultural effect as indicated by this paper title and conclusion.

There seems to be a small difference regarding gender in the interviews where overall the male participants seemed more positive toward Hugvie. Both culturally and in terms of gender it might seem socially inappropriate in Denmark for men to use Hugvie, as some of the participants also expressed, to the point that soft toys may be seen as feminine or childish things. Therefore, it is interesting that it did make a positive difference for most of the male participants – using Hugvie did make them feel more comfortable according to their comments after the sessions. In the previous Japanese experiment participants, who were all female, have not mentioned social acceptance regarding usage of Hugvie, but most seemed positive toward Hugvie.

Although there may be some cultural differences, it is difficult to say whether the reason is that Japanese people tend to be more polite and wrap things up, or if they are just more positive toward the Hugvie experience in general. The Danish participants seemed to give their honest opinions toward Hugvie. One man in particular said that it would be taboo for men to use it because of its toy-like appearance for him, and the women from the group interview raised the question of whether it could be acceptable for men to use Hugvie. We did not have the same comparative conditions such as gender, both male and female, in Japan. The Japanese experiment did not use the STAI questionnaire, so we need to conduct the experiment in Japan again. In such same conditions, we have to carry forward cross-culturally comparative experiments in our future work.

The medium could not be effective for everybody, but we see a development in attitude toward Hugvie, for instance, in a group interview. We had three nurses, two from phone-group and one from Hugvie-group, share their experiences with and without Hugvie and their thoughts prior to the experiment. Hereafter they could all see, touch and hold Hugvie while discussing their viewpoints on it. The nurses started giving Hugvie a gender instead of just calling it a “thing” or “it,” Hugvie became him. This could indicate a form of closeness.

Prior to the experiments, they were all skeptical of Hugvie and the intentions for usage, thinking that this artificial thing would not be able to replace the warm hands of a human when considering their field. But after sitting with it they had Hugvie associated with a child or an animal (penguin) because of its size and shape. One even mentioned it felt like sitting with her grandchild because of the warmth Hugvie provided although the feeling was too artificial in the start and therefore it could use more softness perhaps a fur cover.

Because of the warmth that one felt touched her heart, they suggested it would be good to use Hugvie for residents at nursing homes. Especially the comfort really could be used in such establishments and for demented people. The color of this Hugvie was bright orange and this was also welcomed as a happy color. Though the nurses approved of Hugvie they still felt it might be limited to certain groups of people and that men might refrain from using it and therefore mainly would be for women.

With this interview, we see the negative or rather skeptical attitude toward Hugvie prior to the experiment turning into an open talk about the usage of it. The three ladies became very open and accepting of the medium, but only one had the actual experience talking through Hugvie. While sitting with Hugvie, though the two others did not share the same experience as the hug-group participant, they developed their opinions in a positive way where they seemed very open minded when listening to her experience.

Many participants described a comfortable feeling and the sense of being with someone, like a pet or a child, when talking through Hugvie, but when we asked whether they would like to use it at home, there was surprisingly few who would even consider it.

A male participant said “If Hugvie should be used outside of the home, it has to become a trend so everyone would use it or else people would think the user would be weird,” “It could be smaller maybe,” “But, I think that there is some meaning to the big size in terms of how calm it makes you, because it actually feels almost like two people sitting and talking. You don’t feel alone.”

Overall when asking about the usage at home, we got comments similar to this – “It is hard to imagine using it in practice, if you had one at home.”

The opinions about who could or could not use Hugvie should be considered according to social norms since these questions and comments are made. This should not be limited to one country, but should be studied cross-culturally because there would possibly be a different opinion and attitude depending on the way people are raised, and which environment they have lived in. Age could also be a factor for the assumption that men would not be able to use Hugvie as standard viewpoints could change depending on the generation and media exposure.

## Conclusion

We found that Hugvie was effective in reducing anxiety for Danes, with significant difference in the questionnaire between baseline and post-encounter scores in the Hugvie group, but no significant differences in the phone group. Essentially, we found that the difference was related to the personal trait openness. Statistically, no significant gender differences were found, but it might be due to the small number of participants in each group. Still there seemed a slight difference in the interviews according to who can use Hugvie or not, but this is more a question of norm than whether it can have a stress reducing effect. We suggest this for further research.

The indication of openness in the results suggest users of the huggable medium to have a certain sensitivity, like active imagination and aesthetic sensitivity, to reach a stress reducing effect from using Hugvie. Participants who score higher on openness and spend time with Hugvie had a greater likelihood of experiencing anxiety reduction.

Although Hugvie resembled an animal, a child or just felt comfortable to sit with for some, others did not like it at all. The participants had a common opinion when it came to whether they could imagine using the communication medium at home; they did not want to use it by themselves, but many suggested care facilities or lonely elderly for primary users. Mainly because they thought it was impractical to use when moving around and having to find it to insert the phone before usage, which could be the reason why immobile people came to mind.

The participants mentioned improvements such as it becoming softer, more mobile/handy and the pocket/head part more stable to make Hugvie more preferable. If they were lonely or demented, it might be more acceptable to use a toy like phone or else it could just be that the Hugvie should be redesigned if it should be introduced to people who are used to a more flexible way of communicating.

There are such limitations as lack of comparison of the Danish study with a Japanese one that investigates the effect of Hugvie in all the same conditions and the cortisol test did not give a clear result, so we should have used other methods like amylase to supplement and other types of hormones. We could have used a larger group to investigate the details of gender difference and the interviews could have touched different mentalities and norms cross-culturally. Social norms related to usage of Hugvie and its cultural relation to personalities needs to be investigated in terms of the acceptability of new media in societies.

In the results, we found that personality matters for the usage of communication media. We suggest that when we apply communication media to people, we should investigate personality traits that could affect the effects of the media, perhaps with a possible matrix of personality and various types of communication media. There are other factors such as gender and cultural differences, which might affect the effects as well and we could look into the components of these factors. This could be tested through experiments with different types of classified communication media such as regular phones, video-conferencing systems, huggable communication media, and various types of social robots – mechanoid, zoomorphic, and humanoid.

## Author Contributions

RY: project leader; LC, and KS: experiment staff; C-CC, MD, and HS: analysis of data; SN: adviser; HI: media inventor.

## Conflict of Interest Statement

The authors declare that the research was conducted in the absence of any commercial or financial relationships that could be construed as a potential conflict of interest.
